# Integrating the Technical, Risk Management and Economic Implications of Animal Disease Control to Advise Policy Change: The Example of Foot-and-Mouth Disease Control in Uruguay

**DOI:** 10.1007/s10393-020-01489-6

**Published:** 2020-10-14

**Authors:** Brian Perry, Karl M. Rich, Hernán Rojas, Jaime Romero, David Adamson, José Eduardo Bervejillo, Federico Fernandez, Alvaro Pereira, Lautaro Pérez, Fernando Reich, Rafael Sarno, Edgardo Vitale, Federico Stanham, Jonathan Rushton

**Affiliations:** 1 Nuffield College of Clinical Medicine, University of Oxford, Oxford, UK; 2 College of Medicine and Veterinary Medicine, University of Edinburgh, Edinburgh, UK; 3 Foresight Modeling and Policy Unit, Policies, Institutions, and Livelihoods, West Africa Regional Office, International Livestock Research Institute (ILRI), Dakar, Senegal; 4 CERES BCA, Santiago, Chile; 5 Sanidad Agropecuaria, Calidad e Inocuidad de los Alimentos, Instituto Interamericano de Cooperación para la Agricultura (IICA), San José, Costa Rica; 6 The Centre for Global Food and Resources (GFAR), The University of Adelaide, Adelaide, Australia; 7 Ministerio de Ganadería, Agricultura y Pesca, Montevideo, Uruguay; 8 Instituto Nacional de Carnes (INAC), Montevideo, Uruguay; 9 Animal Health and Food Systems Economics, University of Liverpool’s Centre of Excellence for Sustainable Food Systems, Institute of Infection and Global Health, University of Liverpool, Liverpool, UK

Countries contemplating a change in their animal disease control policy face a variety of considerations, particularly in circumstances in which disease status, and the use (or not) of vaccines to control or minimise disease risk, has major implications for international trade. Foot-and-mouth disease (FMD) exemplifies these trade-offs, and is particularly important in South America, where FMD virus circulation has declined and appears limited to certain regions. As a result, opportunities for higher-value exports in sustainably produced pasture-fed beef and lamb are growing.

Uruguay is arguably at the forefront of these developments. It is renowned for an efficient livestock production base, high standards of animal health, and a pasture-based, extensive feeding system. Uruguay exports over four per cent of the world’s fresh and frozen meat (https://oec.world/en/profile/country/ury/), and in 2018, 70% of these exports went to China (Joseph 2019). Parts of neighbouring countries such as Brazil, Argentina and Paraguay share the advantages of pasture-based feeding, and also aspire to sell to more diverse international markets.

Export market access for all these countries depends on the successful control of FMD. A country’s FMD status (whether endemic, free with vaccination, or free without vaccination) has implications for market access and prices, and these depend on trading partners’ willingness to accept different levels of risk. Some of the highest value markets for beef, such as Japan and Korea, only allow imports from the very small subset of countries that are FMD-free without vaccination against FMD (Rich and Winter-Nelson 2007). Uruguay and its neighbours are contemplating new FMD policy measures, including the cessation of blanket vaccination, in order to improve the quality, quantity and diversity of their markets. This will also contribute to the broader hemispheric aspirations of PHEFA (Hemispheric Foot and Mouth Disease Control Programme 2011–2020), together with the countries of South America and Panama, to eradicate FMD under the coordination of PANAFTOSA.^1^

In May 2019, the Uruguayan Ministry of Livestock, Agriculture and Fisheries (MGAP), the Instituto Nacional de Investigación Agropecuaria (INIA), and the Instituto Nacional de Carnes (INAC) jointly commissioned an independent evaluation of the implications of moving to a no FMD vaccination policy in the country, and to assess the technical, risk management and economic implications of any such change. The authors undertook this study, and in-country meetings and workshops were conducted in May, June, August and October 2019. Here, we present the study results and the broader implications of such interdisciplinary team studies to underlie animal health policy change in other counties and for other trade-related diseases.

## Methodology

The analysis considered three new policy approaches to capitalise further on Uruguay’s continued FMD freedom. These were:

- Elimination of FMD vaccination (termed NO vaccination).
- Elimination of FMD vaccination accompanied by an enhancement of animal health service capacity (NO vaccination PLUS). This was designed to strengthen various components of veterinary services such as biosecurity, surveillance and service management.
- Maintenance of an annual vaccination programme, accompanied by an enhancement of animal health service capacity (Vaccination PLUS).

For each alternative policy option, an assessment was made on the impact of four different potential FMD scenarios (Table 1). The most likely scenario, given the data and information received, was no FMD, and this has been taken into account in the subsequent cost–benefit analysis (CBA). A range of mitigation measures was developed through team discussion for each control policy and scenario, with costs developed based on the MGAP Contingency Plan^2^ and the adjustments that can be made at the time of the contingency.

**Table 1:** Matrix of alternative FMD policy scenarios.

Prevention, surveillance, management and control policy for FMD freedom	Potential FMD scenario			
	No FMD in the country or neighbouring countries	FMD outbreak in neighbouring country	Low magnitude FMD outbreak in Uruguay	High magnitude FMD outbreak in Uruguay
Current: annual FMD vaccination				
No vaccination				
No vaccination PLUS				
Vaccination PLUS				

**Figure 1:**
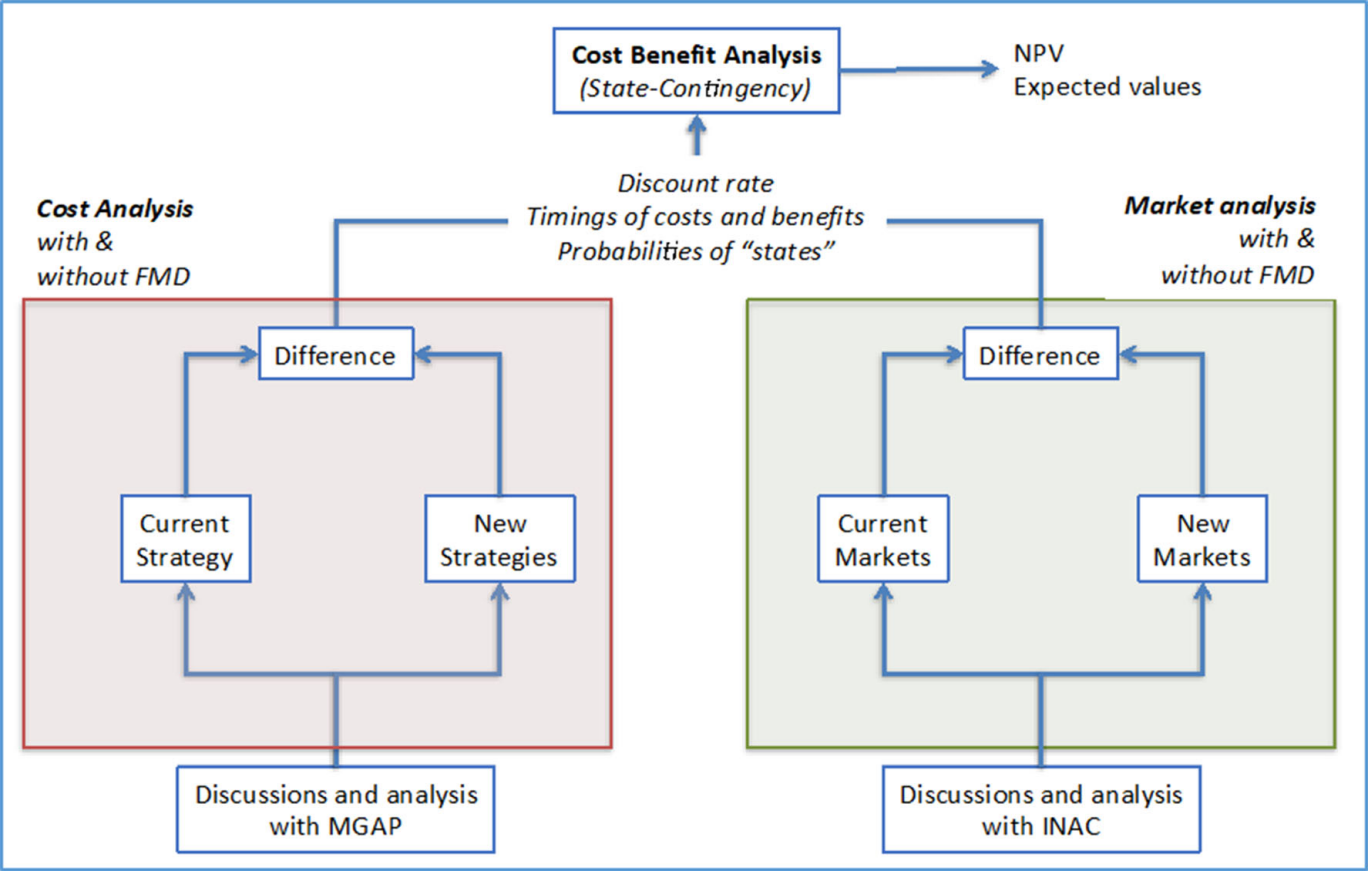
Summary of the cost–benefit analysis performed.

For each of the policy comparisons, an assessment of prospective market access benefits and animal health service costs were derived, which were incorporated into a CBA (Fig. 1). Market access benefits were derived based on an exhaustive analysis of the prospective returns associated with obtaining higher prices and market share and access for specific cuts that would be possible under the cessation of annual FMD vaccination. This includes access for offals and certain bone-in cuts to East Asian and European markets which are currently closed to Uruguay. Additionally, implicit benefits to being FMD-free without vaccination were explored with regards to the speed of regaining market access after a FMD outbreak. There is evidence that this is faster for a country free with no vaccination than for a country that is free with vaccination.

Information on the costs of new mitigation measures was generated through data collected from MGAP, private sector stakeholders and secondary sources. Gaps were filled with consultation within the team and with key public and private sector informants in the country. These results were presented at three different workshops held in Uruguay and were validated during that period.

Information on the costs and benefits for each policy and for each FMD scenario were calculated over a 20-year period with a discount rate of 8%. To obtain an understanding of the relative value of the different policies given the uncertainty of FMD status, a state contingent approach (Adamson and Loch 2020) was adopted that allows an assignment of probabilities for each policy and FMD status to be combined with the cost–benefit analysis results.

## Results

The market analysis indicates that if Uruguay changes to a NO vaccination policy, there will be opportunities to increase income from its traded meat and livestock products. In general, direct market access benefits are positive but fairly modest, with short-term benefits estimated at over US$3 million, medium-term benefits of nearly US$6 million, and longer-term benefits of just over US$25 million annually.

While the new market opportunities are not extensive, and likely to generate relatively modest gains in export revenues, it is important to recognise that the analysis considered Uruguay in isolation from the other Mercosur countries (Argentina, Brazil and Paraguay). A Mercosur-wide cessation of FMD vaccination would undoubtedly accelerate price competition in both East Asian markets such as Japan and South Korea, as well as in China, as supplying countries would have even more flexibility in maximising carcass value through greater market access of different cuts. However, there may also be important “first-mover” advantages in accessing such markets, in terms of securing relationships and supply chains. Given that Uruguay already has inroads in these target countries and a good reputation on global markets for traceability and reliability, this suggests that there will be potential benefits from accessing these markets first through a more aggressive approach towards market access without the use of FMD vaccination.

The implicit benefits of NO vaccination by the faster re-opening of high value markets in the event of an FMD outbreak are larger. Taking the top 70% of traded products by value (US$1.24 billion of trade) as a point of comparison, we estimate that the likely value of annual exports would be US$89 million less under the Vaccination and Vaccination PLUS strategies in the event of an FMD outbreak, as compared to the NO vaccination case. Under alternative scenarios where domestic prices fall in response to the glut of goods into the domestic market, these differences widen. This suggests positive benefits from the faster re-opening of markets under a NO vaccination policy.

The cost analysis of the current FMD policy (Vaccination) estimated that Uruguay currently spends US$36.9 million on 11 different management components. These costs are split equally between the public and private sectors, and half of the costs are due to FMD vaccination. In the NO vaccination policy, annual costs are estimated to fall to US$18.8 million, but the associated risk analysis undertaken indicates that this policy would also increase the risk of FMD introduction, exposure and dissemination. Moving to NO vaccination will therefore require parallel investments in strengthened veterinary services. The NO vaccination PLUS strategy would cost less than the current FMD vaccination (as there is no cost of vaccine purchase and deployment), and is estimated to cost US$30.2 million, a saving compared to the current policy (Vaccination). The risk analysis for this option indicates that it would also reduce risks of an FMD introduction, exposure and dissemination event.

By contrast, the alternative policy of Vaccination PLUS would increase the annual costs of the programme to US$44.7 million, and also reduce the risk of FMD incursions. The overall risk of FMD was estimated to be very similar to the less expensive NO vaccination PLUS, and would provide no additional market access benefits.

It was recognised that the costs of responses to deal with an FMD incursion, be it an outbreak in a neighbouring country or within Uruguay, would be different between the policies. Costs for the NO vaccination PLUS strategy with a scenario of an outbreak in a neighbouring country were predicted to be lower than other policies, but higher for scenarios where there were outbreaks in Uruguay itself.

The net present value (NPV) results are presented in Table 2. The cost–benefit analysis (with sensitivity analysis on market access, discount rates and varying levels of risk of FMD risk) demonstrated that policies with no annual vaccination were superior and that this result was robust. The savings for these two options (NO vaccination and NO vaccination PLUS) provide a positive cash flow in all of the 20-year evaluation period. On the other hand, the vaccination PLUS strategy always generates a negative cash flow for the cost–benefit analysis. Hence both the internal rate of return and benefit–cost ratios provide no guidance to the analysis.

**Table 2:** Summary of the NPVs of the comparisons between the current strategy with no vaccination, no vaccination PLUS and vaccination PLUS under different disease scenarios with a discount rate of 8%.

Strategy	US$ millions				
	No FMD risk and no FMD present	Increased risk in neighbouring countries	A small scale outbreak in Uruguay	A large scale outbreak in Uruguay	Expected NPV
No vaccination	201.9	− 210.8	− 186.4	− 586.0	194.5
No vaccination PLUS	90.8	− 368	− 397.8	− 662.4	85.6
Vaccination PLUS	− 75.9	− 794	− 1256.8	− 1986.4	− 86.1

## Study Implications

1. Implications for Uruguay

Given current estimated levels of FMD risk, a NO vaccination policy for Uruguay is potentially economically profitable for Uruguay. The sensitivity analysis indicates that this superiority is robust across different assumptions of market access, FMD risk or presence, discount rate and the size of a major outbreak. Additional benefits may exist in sheep meat, dairy products, and the export of live animals, among others, which were not addressed in the market analysis.

Our analysis further demonstrates benefits from switching from an animal health system focused on delivering annual FMD vaccinations to one on strengthening surveillance, improving attention to the general health and welfare needs of livestock, and improving market access for environmentally sustainable products. These benefits accrue to a majority of the people across the livestock sector and society as a whole (see Table 3).

**Table 3:** Winners and losers from a change in vaccination strategy across the food system and society.

Part of the industry	Losers	Neutral	Winners
Supply industry	Pharmaceutical companies producing and distributing FMD vaccines Veterinarians delivering FMD vaccination		Veterinarians with all round healthcare practices
Production	Loss of incidental contributions by veterinary services to farms during vaccination process		Producers with other animal health problems Producers with links to specific markets
Marketing			Companies interested in expanding markets
Processing			Companies with the ability to place products in new markets
Consumers	Consumers of specific products	Majority of consumers	
General			The economy Other exporters of agricultural production

2. Implications for analysing national and regional animal health policy in other countries.

Adapting, changing and renewing animal health policies are challenging processes, particularly when they have trade-offs and broader regional and wider international repercussions. We believe that this is the first such multi-disciplinary analysis of the socioeconomic, logistical and risk factors affecting disease control policy change in Latin America, and it arguably provides a model which could be used by other countries for FMD, for other diseases affecting trade in livestock and livestock commodities, as well as for the broader aspirations of PHEFA. The move from a vaccination-orientated system to a more holistic animal health policy will require training, education and investment (Rojas and Romero 2017). Importantly, it should not be taken as an opportunity to cut animal health budgets, but rather to invest in the animal health systems of the future in a broader and more sustainable manner.

3. The role of integrating epidemiological and economic data and analysis in supporting policy change options.

The value of an integrated epidemiological-economic analysis has long been recognised (see for example Perry et al. 2001, 2013; Rich and Winter-Nelson 2007; Rushton et al. 2009; Rich and Perry 2011; Randolph et al. 2002), but often remain as academic exercises. We present here a practical example designed to influence policy, and one which evolved through teamwork between the designers and implementers.

4. Sustainability issues

The beef sector has come under increasing pressure from the environmental community and others for its contributions to greenhouse gas emissions and global warning (Steinfeld et al. 2006; Gerber et al. 2013). At the same time, there is considerable heterogeneity in production systems, with the extensive systems used in South America (and promoted by Uruguay in its value proposition to consumers), and the silvo-pastoral systems utilised in Central America and Colombia, offering a more environmentally friendly and sustainable means of production (Cuartas et al. 2014; Resende et al. 2020). Improved FMD control at continental level offers greater access to “greener” meat and potentially a basis to move competition away from price towards more intangible attributes associated with production systems. This could expand the benefits that the beef sector and its value chains provide to economic development and environmental preservation within South America.

5. Public–Private Partnerships

Public–private partnerships (PPPs) enable the development of animal health services, policies and trade to a scale, quality or degree of geographic penetration that is unachievable by the public sector alone. The partnership between the three Uruguayan bodies commissioning this study presents an impressive model of a PPP in the field of animal health and trade. The FMD programme is co-financed by both public and private sectors, and this study provided a means to bring together the different stakeholders in each group to debate the strengths and weaknesses of the system in place, and to vision new disease control and livestock trading opportunities. It will be important to build on this PPP base in confronting new animal health challenges, and ensure that it is not compromised in a No vaccination policy.

## Footnotes

1 Pan American Foot-and-Mouth Disease Centre (PANAFTOSA) of the Pan American Animal Health Organisation (PAHO).

2 Foot-and-Mouth Disease Contingency Plan and Manual. Year 2005. SECTION 11. "Health Emergency Manual and Guide", MGAP 2015 and Handbook of Health Emergency Procedures for FMD, MGAP, 2016.
